# Convergence of batch gradient learning with smoothing regularization and adaptive momentum for neural networks

**DOI:** 10.1186/s40064-016-1931-0

**Published:** 2016-03-08

**Authors:** Qinwei Fan, Wei Wu, Jacek M. Zurada

**Affiliations:** School of Science, Xi’an Polytechnic University, Xi’an, 710048 People’s Republic of China; School of Mathematical Sciences, Dalian University of Technology, Dalian, 116024 People’s Republic of China; Department of Electrical and Computer Engineering, University of Louisville, Louisville, KY 40292 USA; Spoleczna Akademia Nauk, 90-011 Lodz, Poland

**Keywords:** Feedforward neural networks, Adaptive momentum, Smoothing $L_{1/2}$ regularization, Convergence

## Abstract

This paper presents new theoretical results on the backpropagation algorithm with smoothing $L_{1/2}$ regularization and adaptive momentum for feedforward neural networks with a single hidden layer, i.e., we show that the gradient of error function goes to zero and the weight sequence goes to a fixed point as *n* (*n* is iteration steps) tends to infinity, respectively. Also, our results are more general since we do not require the error function to be quadratic or uniformly convex, and neuronal activation functions are relaxed. Moreover, compared with existed algorithms, our novel algorithm can get more sparse network structure, namely it forces weights to become smaller during the training and can eventually removed after the training, which means that it can simply the network structure and lower operation time. Finally, two numerical experiments are presented to show the characteristics of the main results in detail.

## Background

A multilayer perceptron network trained with a highly popular algorithm known as the error back-propagation (BP) has been dominating in the neural network literature for over two decades (Haykin 2008). BP uses two practical ways to implement the gradient method: the batch updating approach that accumulates the weight corrections over the training epoch before performing the update, while the online learning approach updates the network weights immediately after each training sample is processed (Wilson and Martinez 2003).

Note that training is usually done by iteratively updating of the weights that reduces error value, which is proportional to the negative gradient of a sum-square error (SSE) function. However, during the training of feedforward neural networks (FNN) with SSE, the weights might become very large or even unbounded. This drawback can be addressed by adding a regularization term to the error function. The extra term acts as a brute-force to drive unnecessary weights to zero to prevent the weights from taking too large values and then it can be used to remove weights that are not needed, and is also called penalty term (Haykin 2008; Wu et al. 2006; Karnin 1990; Reed 1993; Saito and Nakano 2000).

There are four main different penalty approaches for BP training: weight decay procedure (Hinton 1989), weight elimination (Weigend et al. 1991), approximate smoother procedure (Moody and Rognvaldsson 1997) and inner product penalty (Kong and Wu 2001).

In the weight decay procedure, the complexity penalty term is defined as the squared norm of the weight vector, and all weights in the multilayer perceptron are treated equally. In the weight elimination procedure, the complexity penalty represents the complexity of the network as function of weight magnitudes relative to a pre-assigned parameter (Reed 1993).

In approximate smoother procedure, this penalty term is used for a multilayer perceptron with a single hidden layer and a single neuron in the output layer. Compared with the earlier methods, it does two things. First, it distinguishes between the roles of weights in the hidden layer and those in the output layer. Second, it captures the interactions between these two sets of weights, however, it is much more demanding in computational complexity than weight decay or weight elimination methods. In Kong and Wu (2001) the inner-product form is proposed and its efficiency in general performance of controlling the weights is demonstrated. Convergence of the gradient method for the FNN has been considered by Zhang et al. (2015, 2009), Wang et al. (2012) and Shao and Zheng (2011).

The convergence of the gradient method with momentum is considered in Bhaya and Kaszkurewicz (2004), Torii and Hagan (2002), Zhang et al. (2006), in Bhaya and Kaszkurewicz (2004) and Torii and Hagan (2002) under the restriction that the error function is quadratic. Inspired by Chan and Fallside (1987), Zhang et al. (2006) considers the convergence of a gradient algorithm with adaptive momentum, without assuming the error function to be quadratic as in the existing results. However, in Zhang et al. (2006), the strong convergence result is based on the assumption that the error function is uniformly convex, which still seems a little intense.

The size of a hidden layer is one of the most important considerations when dealing with real life tasks using FNN. However, the existing pruning methods may not prune the unnecessary weights efficiently, so how to efficiently simplify the network structure becomes our main task.

Recently, considerable attention has been paid to the sparsity problems and a class of regularization methods was proposed which take the following form:

1 $$\begin{aligned} \min \left\{ \frac{1}{n}\sum _{i=1}^{n}l(y_i, h(x_i))+\lambda \Vert h\Vert _k\right\} \end{aligned}$$

where $l(\cdot ,\cdot )$ is a loss function, $(x_i,y_i)_{i=1}^n$ is a data set, and $\lambda$ is the regularization parameter. When *h* is in the linear form and the loss function is square loss, $\Vert h\Vert _k$ is normally taken as the norm of the coefficient of linear model.

For $k=0$, (1) becomes $L_0$ regularization and can be understood as a penalized least squares with penalty $\Vert h\Vert _0$, which yields the most sparse solutions, but for large data analysis it faces the problem of combinatory optimization (Davis 1994; Natarajan 1995). In order to deal with such difficulty, Tibshirani (1996) proposed $L_1$ regularization where $k=1$ and $\Vert h\Vert _1$ is the $L_1$ norm of n dimensional Euclidean space $R^n$, which just needs to solve a quadratic programming problem but is less sparse than the $L_0$ regularization. At the same time Donoho (1995, 2005) proved that under some conditions the solutions of the $L_0$ regularizer are equivalent to those of the $L_1$, so the hard NP optimization problem can be avoided in the $L_1$ regularizer. In order to find a new regularizer which is more sparse than the $L_1$ regularizer while it is still easier to be solved than the $L_0$ regularizer, in Xu et al. (2010) a modified $L_{1/2}$ regularizer is proposed of the following form:

2 $$\begin{aligned} \hat{\beta }_{L_{{\frac{1}{2}}}}=arg min \left\{ \frac{1}{n}\sum _{i=1}^{n}(Y_i-X_i^T\beta )^2+\lambda \sum _{i=1}^{p}|\beta _i|^{\frac{1}{2}} \right\} \end{aligned}$$

where $$\lambda$$ is the tuning parameter. As shown in Xu et al. (2010), $$L_{1/2}$$ regularizer has a nonconvex penalty and possesses many promising properties such as unbiasedness, sparsity, oracle properties and can be taken as a representative of the $$L_r$$$$(0 < r < 1)$$ regularizer. Recently, we develop a novel method to prune FNNs through modify the usual $$L_{1/2}$$ regularization term by smoothing technique. The new algorithm not only removes the oscillation of the gradient value, but also get better pruning, namely the final weights to be removed are smaller than those produced through the usual $$L_{1/2}$$ regularization (Wu et al. 2014; Fan et al. 2014).

The focus of this paper is on extension of $L_{1/2}$ regularization beyond its basic concept though its augmentation with a momentum term. Also, there are some other applications of FNNs for optimization problems, such as the generalized gradient and recurrent neural network methods shown as Liu et al. (2012) and Liu and Cao (2010)

It is well known that a general drawback of gradient based BP learning process is its slow convergence. To accelerate learning, a momentum term is often added (Haykin 2001; Chan and Fallside 1987; Qiu et al. 1992; Istook and Martinez 2002). By adding momentum to the update formula, the current weight increment is a linear combination of the gradient of the error function and the previous weight increment. As a result, the updates respond not only to the local gradient but also to recent gradient in the error function. Selected reports discuss the NN training with momentum term in the literature (Torii and Hagan 2002; Perantonis and Karras 1995; Qian 1999).

As demonstrated in Torii and Hagan (2002), there always exists a momentum coefficient that will stabilize the steepest descent algorithm, regardless of the value of the learning rate (we will define it below). In addition, it shows how the value of the momentum coefficient changes the convergence properties. Momentum acceleration, its performance in terms of learning speed and scalability properties is evaluated and found superior to the performance of reputedly fast variants of the BP algorithm in several benchmark training tasks in Perantonis and Karras (1995). Qian (1999) shows that in the limit of continuous time, the momentum parameter is analogous to the mass of Newtonian particles that move through a viscous medium in a conservative force field.

In this paper, a modified batch gradient method with smoothing $L_{1/2}$ regularization penalty and adaptive momentum algorithm (BGSAM) is proposed. It damps oscillations present in the $L_{1/2}$ regularization and in the adaptive momentum algorithm (BGAM). In addition, without the requirement that the error function is quadratic or uniformly convex, we present a comprehensive study of the weak and strong convergence for BGSAM which offers an effective improvement in real life application.

The rest of this paper is arranged as follows. The algorithm BGSAM is described in “Batch gradient method with smoothing *L*_1/2_ regularization and adaptive momentum (BGSAM)” section. In “Convergence results” section, the convergence results of BGSAM are presented, and the detailed proofs of the main results are stated in the “Appendix”. The performance of BGSAM is compared to BGAM and the experimental results shown in “Numerical experiments” section. Concluding remarks are in “Conclusions” section.

## Batch gradient method with smoothing $L_{1/2}$ regularization and adaptive momentum (BGSAM)

### Batch gradient method with $L_{1/2}$ regularization and adaptive momentum (BGAM)

Here and below, some definitions and notations used in e.g. Wu et al. (2006), Shao and Zheng (2011), and Wu et al. (2006), Shao and Zheng (2011) have been re-defined and used without repeatedly citing the references. We consider a FNN with three layers, and we denote the numbers of neurons of the input, hidden and output layers by *p*, *q* and 1, respectively. Suppose that $\{\xi ^j,O^j\}_{j=1}^J\subset R^p\times R$ is the given set of *J* training samples. Let $w_0=(w_{10},w_{20},\ldots , w_{q0})^T\in R^q$ be the weight vector between the hidden units and the output unit, and $w_i=(w_{i1},w_{i2},\ldots , w_{ip})^T\in R^p$ be the weight vector between the input units and the hidden unit *i*$(i=1,2,\ldots ,q)$. To simplify the presentation, we combine the weight vectors, and write $W=(w_0^T,w_1^T,\ldots ,w_q^T)^T\in R^{q+pq}$ and we define a matrix $V=(w_1,w_2,\ldots , w_q)^T\in R^{q\times p}$. We also define a vector function $G:R^q\rightarrow R^q$, for $x=(x_1,x_2,\ldots , x_q)^T\in R^q$

3 $$\begin{aligned} G(x)=(g(x_1),g(x_2),\ldots , g(x_q))^T. \end{aligned}$$

Let $g: R\rightarrow R$ be a given transfer function for the hidden and output nodes, which is typically, but not necessarily, a sigmoid function. Then for each input $\xi \in R^p$, the actual output vector of the hidden layer is $G(V\xi )$ and the final output of the network is

4 $$\begin{aligned} g(w_0\cdot G(V\xi )). \end{aligned}$$

For a fixed *W*, the output error function with the $L_{1/2}$ regularization penalty term is

5 $$\begin{aligned} E(W) & = {\frac{1}{2}}\sum _{j=1}^J(O^j-g(w_0\cdot G(V \xi ^j)))^2+\lambda \sum _{i=1}^q\sum _{k=0}^p|w_{ik}|^{\frac{1}{2}}\nonumber \\ & = \sum _{j=1}^Jg_j(w_0\cdot G(V \xi ^j))+\lambda \sum _{i=1}^q\sum _{k=0}^p|w_{ik}|^{\frac{1}{2}} \end{aligned}$$

where $g_j(t):={\frac{1}{2}}(O^j-g(t))^2$, $j=1,2,\ldots ,J$, $t\in R$, $\lambda >0$ is the penalty coefficient, and $|\cdot |$ denotes the absolute value. The gradient of the error function is

6 $$\begin{aligned} E_W(W)=(E_{w_{0}}^T(W), E_{w_{1}}^T(W), \ldots , E_{w_{q}}^T(W))^T \end{aligned}$$

where

$$\begin{aligned} & E_{w_{0}}^T(W)=(E_{w_{10}}(W), E_{w_{20}}(W),\ldots , E_{w_{q0}}(W))\\ & E_{w_{1}}^T(W)=(E_{w_{11}}(W), E_{w_{12}}(W), \ldots , E_{w_{1p}}(W))\\ & E_{w_{2}}^T(W)=(E_{w_{21}}(W), E_{w_{22}}(W), \ldots , E_{w_{2p}}(W))\\ & \cdots \\ & E_{w_{q}}^T(W)=(E_{w_{q1}}(W), E_{w_{q2}}(W), \ldots , E_{w_{qp}}(W)) \end{aligned}$$

The gradient of the error function with respect to $w_{i0}$ and $w_{ik}$ are, respectively, given by

7 $$\begin{aligned} E_{w_{i0}}(W) & = \sum _{j=1}^Jg'_{j}(w_0\cdot G(V \xi ^j))g(w_i \xi ^j)+\frac{\lambda sgn(w_{i0})}{2|w_{i0}|^{\frac{1}{2}}}\end{aligned}$$

8 $$\begin{aligned} E_{w_{ik}}(W) & = \sum _{j=1}^Jg'_{j}(w_0\cdot G(V \xi ^j))w_{i0}g'(w_i\cdot \xi ^j) \xi _k^j+\frac{\lambda sgn(w_{ik})}{2|w_{ik}|^{\frac{1}{2}}} \end{aligned}$$

where $$i=1,2,\ldots ,q,$$ and $$k=1,2,\ldots ,p$$.

The detailed BGAM algorithm is presented as follows. We denote $W^{n+1}=W^n+\Delta W^n,~ n=0,1,2,\ldots$, starting from an arbitrary initial value $W^0$ and $W^1$, and the weights $\{W^n\}$ are updated iteratively by

9 $$\begin{aligned} \Delta W^n=-\eta E_W(W^n)+\alpha _W^n\Delta W^{n-1}, ~~~~~ n=0,1,2,\ldots \end{aligned}$$

The learning rate is assumed constant and satisfies $\eta >0$, and $\alpha _W^n=(\alpha _{w_0}^n,\alpha _{w_1}^n,\ldots ,\alpha _{w_q}^n)$ is the momentum coefficient vector of the n-th training. It consists of coefficients $\alpha _{w_{i}}^n$ for each $\Delta w_{ik}^n (i=1,2,\ldots ,q,~k=1,2,\ldots ,p)$, and $\alpha _{w_{0}}^n$ for each $\Delta w_{i0}^n (i=1,2,\ldots ,q)$. Similar to Shao and Zheng (2011), for every $\alpha _{w_i}^n$, after each training epoch it is chosen as

10 $$\begin{aligned} \alpha _{w_{i}}^n={\left\{ \begin{array}{ll} \alpha \cdot \frac{-\eta E_{w_{i}}(W^n)\cdot \Delta w_{i}^{n-1}}{\Vert \Delta w_{i}^{n-1}\Vert ^2}, &{}\quad if~E_{w_{i}}(W^n)\cdot \Delta w_{i}^{n-1}<0\\ 0,&{}\quad otherwise \end{array}\right. } \end{aligned}$$

where $\alpha \in (0,1)$ is the momentum factor. Compared with the traditional algorithm, the BGAM has better pruning performance, but we notice that this usual $L_{1/2}$ regularization term used in this part involves in absolute values and it is not differentiable at the origin, which will cause difficulty in the convergence analysis. More importantly, it causes oscillations of the error function and the norm of gradient. In order to overcome these drawbacks we improved the BGAM algorithm as follows:

### Smoothing $L_{1/2}$ regularization and adaptive momentum (BGSAM)

This section introduces a modified algorithm with smoothing $L_{1/2}$ regularization and adaptive momentum term. The network structure is the same as the description in part of last subsection (BGAM). We modify the usual $L_{1/2}$ regularization term at the origin (i.e. we replace the absolute values of the weights by a smooth function in a neighborhood of the origin). Then we use a smooth function *f*(*x*) to approximate |*x*|. We get the following error function with a smoothing $L_{1/2}$ regularization penalty term:

11 $$\begin{aligned} E(W) & = {\frac{1}{2}}\sum _{j=1}^J(O^j-g(w_0\cdot G(V \xi ^j)))^2+\lambda \sum _{i=1}^q\sum _{k=0}^pf(w_{ik})^{\frac{1}{2}}\nonumber \\ & = \sum _{j=1}^Jg_j(w_0\cdot G(V \xi ^j))+\lambda \sum _{i=1}^q\sum _{k=0}^pf(w_{ik})^{\frac{1}{2}} \end{aligned}$$

where $g_j(t):={\frac{1}{2}}(O^j-g(t))^2$, $j=1,2,\ldots ,J$, $t\in R$, $\lambda >0$ is the penalty coefficient. Here, by smoothing we mean that, in a neighborhood of the origin, we replace the absolute values of the weights by a smooth function of the weights. For definiteness and simplicity, we choose *f*(*x*) as a piecewise polynomial function such as:

12 $$\begin{aligned} f(x)={\left\{ \begin{array}{ll} |x|, & \quad if~|x|\ge a\\ -\frac{1}{8a^3}x^4+\frac{3}{4a}x^2+\frac{3}{8}a,&\quad if~|x|<a \end{array}\right. } \end{aligned}$$

where *a* is a small positive constant. and $|\cdot |$ denotes the absolute value. Then, from the definition of *f*(*x*) immediately yields

$$\begin{aligned} f(x)\in \left[ \frac{3}{8}a, +\infty \right) , \quad f'(x)\in [-1,1], ~~f''(x)\in \left[ 0,\frac{3}{2a}\right] \end{aligned}$$

The gradient of the error function with respect to *W* as in (6), and the gradients of the error function with respect to $w_{i0}$ and $w_{ik}$ are then as follows:

13 $$\begin{aligned} E_{w_{i0}}(W) & = \sum _{j=1}^Jg'_{j}(w_0\cdot G(V \xi ^j))g(w_i \cdot \xi ^j) +\lambda \frac{f'(w_{i0})}{2f(w_{i0})^{\frac{1}{2}}} \end{aligned}$$

14 $$\begin{aligned} E_{w_{ik}}(W) & = \sum _{j=1}^Jg'_{j}(w_0\cdot G(V \xi ^j))w_{i0}g'(w_i\cdot \xi ^j) \xi _k^j +\lambda \frac{f'(w_{ik})}{2f(w_{ik})^{\frac{1}{2}}} \end{aligned}$$

where $$i=1,2,\ldots ,q,~~k=1,2,\ldots ,p$$.

For BGSAM algorithm, we denote $W^{n+1}=W^n+\Delta W^n,~ n=0,1,2,\ldots$. Starting with an initial value $W^0$ and $W^1$, the weights $\{W^n\}$ are updated iteratively by

15 $$\begin{aligned} \Delta W^n=-\eta E_W(W^n)+\alpha _W^n\Delta W^{n-1}, \quad n=0,1,2,\ldots \end{aligned}$$

Here the learning rate $\eta$, the momentum coefficient vector of the n-th training $\alpha _W^n$ and other coefficients are the same as the description of algorithm BGAM. For each $\alpha _{w_i}^n$, after each training epoch it is chosen as (10).

## Convergence results

The following assumptions are needed to introduce the relating convergence theorems of BGSAM.

(*A*1): |*g*(*t*)|, $|g'(t)|$, $|g''(t)|$ are uniformly bounded for $t\in R$.
(*A*2): There exists a bounded region $\Omega \subset R^n$ such that $\{w_0^n\}_{n=0}^\infty \subset \Omega$.
(*A*3): $0<\eta <\frac{1}{(M\lambda +C_1)(1+\alpha )^2}$, where $M=\frac{\sqrt{6}}{4\sqrt{a^3}}$ and $C_1$ is a constant defined in (16) below.

Assume conditions (*A*1)–(*A*2) is valid. Then there are some positive constants $C_1$–$C_5$ such that

16 $$\begin{aligned} C_1 & = J(1+C_2)C_3 \max \{C_2^2, C_5^2\}+{\frac{1}{2}}J(1+C_2)C_3 +{\frac{1}{2}}JC_3^2C_4^2C_5,\nonumber \\ C_2 & = \max \left\{\sqrt{q}C_3,(C_3C_4)^2\right\},\nonumber \\ C_3 & = \max \left\{\sup \limits _{t\in R}|g(t)|,\sup \limits _{t\in R}|g'(t)|,\sup \limits _{t\in R}|g''(t)|, \sup \limits _{t\in R,1\le j\le J}|g'_j(t)|,\sup \limits _{t\in R,1\le j\le J}|g''_j(t)|\right\},\nonumber \\ C_4 & = \max \limits _{1\le j\le J}\Vert \xi ^j\Vert ,~~~ C_5=\sup \limits _{n\in N}\Vert w_0^n\Vert . \end{aligned}$$

### **Theorem 1**

*If assumptions *$(A1){-}(A3)$* are valid for any arbitrary initial value *$W^0$* and *$W^1$*, the error function be defined by (*1*), and let the learning sequence 1*$\{W^n\}$* be generated by the iteration algorithm (*15*), then we have the following convergence*

(i) $\lim \nolimits _{n\rightarrow \infty }E_W(W^n)=0$*. Moreover, (A4) if there exists a compact set *$\Phi$* such that *$W^n\in \Phi$* and the set *$\Phi _0=\{W\in \Phi : E_W(W)=0\}$* contains finite points also holds, then we have the following convergence*
(ii) $$\lim \nolimits _{n\rightarrow \infty }(W^n)=W^*,$$* where *$$W^*\in \Phi _0$$.

## Numerical experiments

This section presents the simulations that verify the performance of BGAM and BGSAM. Our theoretical results are experimentally verified with the 3-bit parity problem, which is a typical benchmark problem in area of the neural networks.

**Fig. 1:**
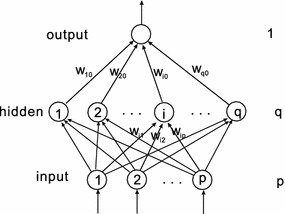
Feedforward neural network with one hidden layer and one output

The two algorithms (BGAM and BGSAM) are implemented by the networks with the structure 5-7-1 (input $p=5$, hidden $q=7$ and output nodes, see Fig. 1). Each of the two algorithms are carried out fifty trials for 3-bit parity problem and then take the mean values, and the termination criterion is that the error is ${<}$1e$-$6 or 3000 iterations. For the network with linear output, we set the transfer function for hidden neurons to be $tansig(\cdot )$ and that for output layer to be $g(t)=t$. For the network with nonlinear output, the transfer functions for both hidden and output neurons are $tansig(\cdot )$. The inputs and the ideal outputs are shown in Table 1.

**Table 1:** 3-bit parity problem

Input				Output	Input				Output
1	1	1	−1	1	1	−1	−1	−1	1
1	1	−1	−1	0	−1	1	1	−1	0
1	−1	1	−1	0	−1	−1	1	−1	1
−1	−1	−1	−1	0	−1	1	−1	−1	1

**Fig. 2:**
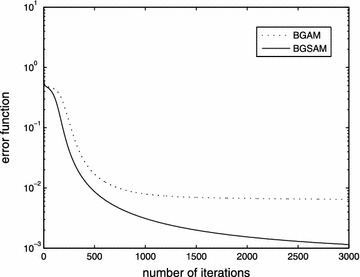
The curve of error function

**Fig. 3:**
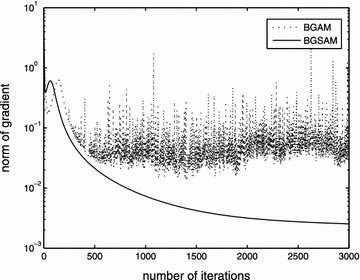
The curve of norm of gradient

**Fig. 4:**
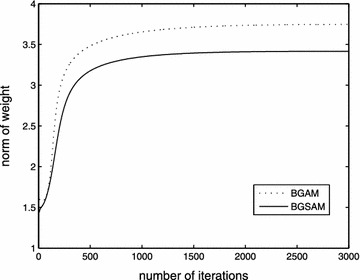
The curve of norm of weight

The performance results of BGAM and BGSAM are shown in the following figures. Figures 2, 3 and 4 present the comparison results for learning rate $\eta$, penalty parameter $\lambda$ and momentum term $\alpha$ with 0.01, 0.0006 and 0.03, respectively.

From Figs. 2 and 3, it can be seen that the error function decreases monotonically and the norm of the gradient of the error function approaches zero as depicted by the convergence theorem, respectively. Also Fig. 3 show us that our modified algorithm overcomes the drawbacks of numerical oscillations, i.e., for BGSAM the norm of gradient curve is much smoother than BGAM. The reason as the following: Since the derivative of |*x*| jumps from $-1$ to $+1$ near the $x=0$, the learning process of *BGAM* will oscillate when a weight $w_{ik}$ is close to zero, whic prevents it from getting further closer to zero. And on the contrary, the derivative of *f*(*x*), which is a smooth approximation of |*x*|, is smooth and equal to zero at the origin, and will not cause any oscillation in the learning process when $w_{ik}$ is close to zero. In the meantime, it can be seen that BGSAM convergence faster than BGAM. Fig. 4 demonstrates that the effectiveness of the algorithm BGSAM in controlling the magnitude of weights is better than BGAM.

## Conclusions

In this paper, the smoothing $L_{1/2}$ regularization term with adaptive momentum is introduced into the batch gradient learning algorithm to prune FNN. First, it removes the oscillation of the gradient value. Second, the convergence results for three-layer FNN are proved under certain relaxed conditions. Third, the algorithm is applied to a 3-bit parity problem and the related results are supplied to support the theoretical findings above. Finally, this new algorithm will also effective for other types neural networks or big data processing.

## Appendix

In the following section three useful lemmas are given for convergence analysis for BGSAM algorithm. For the sake of description, we introduce the following notations:

17 $$\begin{aligned} G^{n,j} & = G(V^n \xi ^j)\nonumber \\ \psi ^{n,j} & = G^{n+1,j}-G^{n,j}\nonumber \\ \Delta w_{i}^n & = w_{i}^{n+1}-w_{i}^n \quad for ~i=1,2,\ldots q\nonumber \\ \Delta w_{0}^n & = w_{0}^{n+1}-w_{0}^n \end{aligned}$$

Using the error function (11) we have

$$\begin{aligned} E\left( W^{n+1}\right) & = \sum _{j=1}^Jg_j\left( w_0^{n+1}\cdot G\left( V^{n+1} \xi ^j\right) \right) +\lambda \sum _{i=1}^q\sum _{k=0}^pf\left( w_{ik}^{n+1}\right) ^{{\frac{1}{2}}}\\ E\left( W^{n}\right) & = \sum _{j=1}^Jg_j\left( w_0^{n}\cdot G\left( V^{n} \xi ^j\right) \right) +\lambda \sum _{i=1}^q\sum _{k=0}^pf\left( w_{ik}^{n}\right) ^{{\frac{1}{2}}} \end{aligned}$$

### **Lemma 1**

(Wu et al. 2010, Lemma 1) *Suppose that*$F:R^Q\rightarrow R$*is continuous and differentiable on a compact set*$D\subset R^Q$*, and that*$\bar{\Omega }=\{x\in D|\frac{\partial F(x)}{\partial x}=0\}$*contains only finite number of points. If a sequence*$\{x^k\}\subset D$*satisfies*

$$\begin{aligned} \lim \limits _{k\rightarrow \infty }\left\| x^{k+1}-x^k\right\| =0, \quad \lim \limits _{k\rightarrow \infty } \left\| \frac{\partial F(x^k)}{\partial x}\right\| =0, \end{aligned}$$

*then, there exists*$x^*\in \bar{\Omega }$*such that*$\lim \nolimits _{k\rightarrow \infty }x_k=x^*$.

The above lemma is crucial for the strong convergence analysis, and it basically follows the same proof as Lemma 1 in Wu et al. (2010). So its proof is omitted.

Now, we give the **proofs of Theorem**1 through the following 4 steps.

**Step 1.** We show the following inequalities holds

18 $$\begin{aligned} \Vert G(x)\Vert & \le \sqrt{q}\sup \limits _{t\in R}|g(t)|\le C_2,\quad x\in R^q \end{aligned}$$

19 $$\begin{aligned} \left\| \psi ^{n,j} \right\| ^2 & \le C_2\sum _{i=1}^q\left\| \Delta w_i^{n}\right\| ^2\nonumber \\ & \le C_2\eta ^2(1+\alpha )^2\sum _{i=1}^q\left\| E_{w_{i}}(W^n)\right\| ^2\quad j=1,2,\ldots ,J. \end{aligned}$$

### *Proof*

With the assumption (*A*1), (16) and the definition of *G*(*x*), it is easy to show that there exists a positive constant $C_2$ such that (18) holds.

By the Lagrange mean value theorem, (*A*1) and (16), we obtain that

20 $$\begin{aligned} \left\| \psi ^{n,j} \right\| ^2= \begin{Vmatrix} \begin{pmatrix} g'(\tilde{t}_{1,j,n})(w_1^{n+1}-w_1^n)\cdot \xi ^j\\ g'(\tilde{t}_{2,j,n})(w_2^{n+1}-w_2^n)\cdot \xi ^j\\ \vdots \\ g'(\tilde{t}_{q,j,n})(w_q^{n+1}-w_q^n)\cdot \xi ^j \end{pmatrix} \end{Vmatrix}^2 \le C_2\sum _{i=1}^q\left\| \Delta w_i^{n}\right\| ^2 \end{aligned}$$

where $$\tilde{t}_{i,j,n}\in R$$$$(1\le i \le q, 1\le j \le J)$$ is between $$w_i^{n}\cdot \xi ^j$$ and $$w_i^{n+1}\cdot \xi ^j$$.

Furthermore, in terms of (9) and (10) we can show that

21 $$\begin{aligned} \Vert \Delta w_{i}^n\Vert & = \left\| -\eta E_{w_{i}}(W^n)+\alpha _{w_{i}}^n\cdot \Delta w_{i}^{n-1}\right\| \nonumber \\ & \le \eta (1+\alpha )\Vert E_{w_{i}}(W^n)\Vert \end{aligned}$$

On the basis of the above inequalities (20) and (21) we immediately have (19). $\square$

**Step 2.** We show the following monotonicity of the sequence $\{E(W^n)\}$

22 $$\begin{aligned} E(W^{n+1})\le E(W^n),\quad n=0,1,2,\ldots \end{aligned}$$

### *Proof*

According to the definition of $w_0^n$ and $\psi ^{n,j}$, we get

23 $$\begin{aligned} w_0^n\psi ^{n,j}=\sum _{i=1}^q w_{i0}^n \left( g\left( w_i^{n+1}\cdot \xi ^j\right) -g\left( w_i^{n}\cdot \xi ^j\right) \right) \end{aligned}$$

By the Taylor mean value theorem with Lagrange remainder to *g*(*t*) at the point $w_i^n\cdot \xi ^j$, we obtain

24 $$\begin{aligned}&g\left( w_i^{n+1}\cdot \xi ^j\right) -g\left( w_i^{n}\cdot \xi ^j\right) \nonumber \\&\quad =g'\left( w_i^{n}\cdot \xi ^j\right) \cdot \Delta w_i^{n}\cdot \xi ^j+{\frac{1}{2}}g''\left( t_{i,j,n}\right) \left( \Delta w_i^{n}\cdot \xi ^j\right) ^2 \end{aligned}$$

where each $t_{i,j,n}$ lies on the segment between $w_i^{n+1}\cdot \xi ^j$ and $w_i^n\cdot \xi ^j$, $i=1,2,\ldots ,q; j=1,2,\ldots ,J$.

Together with (10) and (14), we get

25 $$\begin{aligned}&\sum _{j=1}^Jg'_j\left( w_0^{n}\cdot G^{n,j}\right) w_0^n\psi ^{n,j}\nonumber \\&\quad =\sum _{i=1}^q\sum _{j=1}^Jg'_j\left( w_0^{n}\cdot G^{n,j}\right) w_{i0}^ng'\left( w_i^{n}\cdot \xi ^j\right) \cdot \Delta w_i^{n}\cdot \xi ^j+\delta _1\nonumber \\&\quad =\sum _{k=1}^p\sum _{i=1}^q \sum _{j=1}^Jg'_j\left( w_0^{n}\cdot G^{n,j}\right) w_{i0}^ng'\left( w_i^{n}\cdot \xi ^j\right) \cdot \Delta w_{ik}^{n}\cdot \xi _k^j+\delta _1\nonumber \\&\quad =\sum _{k=1}^p\sum _{i=1}^qE_{w_{ik}}(W^n)\cdot \Delta w_{ik}^{n}-\lambda \sum _{k=1}^p\sum _{i=1}^q\frac{f\left( w_{ik}^n\right) \cdot \Delta w_{ik}^n}{2f\left( w_{ik}^n\right) ^{{\frac{1}{2}}}}+\delta _1\nonumber \\&\quad =\sum _{i=1}^qE_{w_{i}}(W^n)\cdot \Delta w_{i}^{n}-\lambda \sum _{k=1}^p\sum _{i=1}^q\frac{f\left( w_{ik}^n\right) \cdot \Delta w_{ik}^n}{2f(w_{ik}^n)^{{\frac{1}{2}}}}+\delta _1\nonumber \\&\quad =\sum _{i=1}^q E_{w_{i}}(W^n)\left( -\eta E_{w_{i}}(W^n)+\alpha _{w_{i}}^n\cdot \Delta w_{i}^{n-1}\right) \nonumber \\&\qquad -\lambda \sum _{k=1}^p\sum _{i=1}^q\frac{f\left( w_{ik}^n\right) \cdot \Delta w_{ik}^n}{2f\left( w_{ik}^n\right) ^{{\frac{1}{2}}}}+\delta _1\nonumber \\&\quad =-\eta \sum _{i=1}^q\left\| E_{w_{i}}(W^n)\right\| ^2+\sum _{i=1}^q \alpha _{w_{i}}^n\cdot E_{w_{i}}(W^n))\cdot \Delta w_{i}^{n-1}\nonumber \\&\qquad -\lambda \sum _{k=1}^p\sum _{i=1}^q\frac{f(w_{ik}^n)\cdot \Delta w_{ik}^n}{2f(w_{ik}^n)^{{\frac{1}{2}}}}+\delta _1 \end{aligned}$$

where $\delta _1={\frac{1}{2}}\sum _{i=1}^q \sum _{j=1}^Jg'_j\left( w_0^{n}\cdot G^{n,j}\right) w_{i0}^ng''\left( t_{i,j,n}\right) \left( \Delta w_i^{n}\cdot \xi ^j\right) ^2$, and $t_{i,j,n}\in R$ is between $w_i^{n}\cdot \xi ^j$ and $w_i^{n+1}\cdot \xi ^j$.

Using (10) and (13), we obtain

26 $$\begin{aligned}&\sum _{j=1}^Jg'_j\left( w_0^{n}\cdot G^{n,j}\right) G^{n,j}\Delta w_0^n\nonumber \\&\quad =\sum _{i=1}^q \sum _{j=1}^Jg'_j\left( w_0^{n}\cdot G^{n,j}\right) g\left( w_i^n\xi ^j\right) \cdot \Delta w_{i0}^n\nonumber \\&\quad =\sum _{i=1}^q \left( E_{w_{i0}}(W^n)-\lambda \frac{f'\left( w_{i0}^n\right) }{2f\left( w_{i0}^n\right) ^{{\frac{1}{2}}}}\right) \cdot \Delta w_{i0}^n\nonumber \\&\quad =E_{w_{0}}(W^n) \Delta w_{0}^n-\lambda \sum _{i=1}^q\frac{f'\left( w_{i0}^n\right) \cdot \Delta w_{i0}^n}{2f\left( w_{i0}^n\right) ^{{\frac{1}{2}}}}\nonumber \\&\quad =E_{w_{0}}(W^n)\left( -\eta E_{w_{0}}(W^n)+\alpha _{w_{0}}^n\cdot \Delta w_{0}^{n-1}\right) \nonumber \\&\qquad -\lambda \sum _{i=1}^q\frac{f'\left( w_{i0}^n\right) \cdot \Delta w_{i0}^n}{2f\left( w_{i0}^n\right) ^{{\frac{1}{2}}}}\nonumber \\&\quad =-\eta \left\| E_{w_{0}}(W^n)\right\| ^2+\alpha _{w_{0}}^n E_{w_{0}}(W^n)\Delta w_{0}^{n-1}\nonumber \\&\qquad -\lambda \sum _{i=1}^q \frac{f'\left( w_{i0}^n\right) \cdot \Delta w_{i0}^n}{2f\left( w_{i0}^n\right) ^{{\frac{1}{2}}}} \end{aligned}$$

Apply the Taylor mean value theorem with Lagrange remainder, we have

27 $$\begin{aligned}&E(W^{n+1})-E(W^{n})\nonumber \\&\quad =\sum _{j=1}^J\left[ g'_j\left( w_0^{n}\cdot G^{n,j}\right) \cdot (w_0^{n+1}\cdot G^{n+1,j}-w_0^{n}\cdot G^{n,j})\right] \nonumber \\&\qquad +\lambda \sum _{i=1}^q\sum _{k=0}^p\left( f\left( w_{ik}^{n+1}\right) ^{{\frac{1}{2}}}-f\left( w_{ik}^{n}\right) ^{{\frac{1}{2}}}\right) +\delta _2\nonumber \\&\quad =\sum _{j=1}^Jg'_j\left( w_0^{n}\cdot G^{n,j}\right) G^{n,j}\Delta w_0^n +\sum _{j=1}^Jg'_j\left( w_0^{n}\cdot G^{n,j}\right) w_0^n\psi ^{n,j}\nonumber \\&\qquad +\lambda \sum _{i=1}^q\sum _{k=0}^p\left( f\left( w_{ik}^{n+1}\right) ^{{\frac{1}{2}}}-f\left( w_{ik}^{n}\right) ^{{\frac{1}{2}}}\right) +\delta _2+\delta _3 \end{aligned}$$

where $\delta _2={\frac{1}{2}}\sum _{j=1}^Jg''_j(s_{n,j})(w_0^{n+1}\cdot G^{n+1,j}-w_0^{n}\cdot G^{n,j})^2$, $\delta _3=\sum _{j=1}^Jg'_j(w_0^{n}\cdot G^{n,j})\Delta w_0^n\psi ^{n,j}$ and $s_{n,j}\in R$ is a constant between $w_0^{n}\cdot G^{n,j}$ and $w_0^{n+1}\cdot G^{n+1,j}$.

Substituting (25) and (26) into (27) and using the Lagrange mean value theorem for *f*(*x*), we have

28 $$\begin{aligned}&E(W^{n+1})-E(W^{n})\nonumber \\&\quad =-\eta \left\| E_{W}(W^n)\right\| ^2-\lambda \sum _{i=1}^q \sum _{k=0}^p \frac{f'\left( w_{ik}^n\right) \cdot \Delta w_{ik}^n}{2f\left( w_{ik}^n\right) ^{{\frac{1}{2}}}}\nonumber \\&\qquad +\left[ \sum _{i=1}^q \alpha _{i}^n E_{w_{i}}(W^n) \cdot \Delta w_{i}^{n-1}+\alpha _{0}^n E_{w_{0}}(W^n) \cdot \Delta w_{0}^{n-1}\right] \nonumber \\&\qquad +\delta _1+\delta _2+\delta _3 +\lambda \sum _{i=1}^q \sum _{k=0}^p\left( f\left( w_{ik}^{n+1}\right) ^{\frac{1}{2}}-f\left( w_{ik}^n\right) ^{\frac{1}{2}}\right) \nonumber \\&\quad \le -\eta \left\| E_{W}(W^n)\right\| ^2+\frac{\lambda }{2} \sum _{i=1}^q \sum _{k=0}^p F''(t_{i,k,n})(\Delta w_{ik})^2\nonumber \\&\qquad +\delta _1+\delta _2+\delta _3 \end{aligned}$$

where $t_{i,k,n}\in R$ is between $w_{ik}^{n}$ and $w_{ik}^{n+1}$.

According to (21) and (28) we can conclude

29 $$\begin{aligned}&E(W^{n+1})-E(W^{n})\nonumber \\&\quad \le -\eta \left\| E_{W}(W^n)\right\| ^2+M\lambda \sum _{i=1}^q \sum _{k=0}^p (\Delta w_{ik})^2+ \delta _1+\delta _2+\delta _3\nonumber \\&\quad =-\eta \left\| E_{W}(W^n)\right\| ^2+M\lambda \left[ \sum _{i=1}^q \Vert \Delta w_{i}\Vert ^2+ \Vert \Delta w_{0}\Vert ^2\right] + \delta _1+\delta _2+\delta _3\nonumber \\&\quad \le -\eta \left\| E_{W}(W^n)\right\| ^2+M\lambda \eta ^2(1+\alpha )^2 \left\| E_{W}(W^n)\right\| ^2 +\delta _1+\delta _2+\delta _3\nonumber \\&\quad \le -\eta (1-\lambda \eta (1+\alpha )^2M)\left\| E_{W}(W^n)\right\| ^2+\delta _1+\delta _2+\delta _3 \end{aligned}$$

Set $M=\frac{\sqrt{6}}{4\sqrt{a^3}}$, and $F(x)\equiv (f(x))^{{\frac{1}{2}}}$. Note that

$$\begin{aligned} F'(x) & = \frac{f'(x)}{2\sqrt{f(x)}}\\ F''(x) & = \frac{2f''(x)\cdot f(x)-[f'(x)]^2}{4[f(x)]^{\frac{3}{2}}}\\ & \le \frac{f''(x)}{2\sqrt{f(x)}}\\ & \le \frac{\sqrt{6}}{2\sqrt{a^3}} \end{aligned}$$

It follows from (16), (18), (19) and the Cauchy-Schwartz inequality that

30 $$\begin{aligned} |\delta _2| & \le \frac{C_3}{2}\sum _{j=1}^J\left( \Delta w_0^{n} G^{n+1,j}+w_0^{n}\psi ^{n,j}\right) ^2\nonumber \\ & \le \frac{C_3}{2}\sum _{j=1}^J\left( C_2\left\| \Delta w_0^{n}\right\| +C_5\left\| \psi ^{n,j}\right\| \right) ^2\nonumber \\ & \le C_6\sum _{j=1}^J\left( \left\| \Delta w_0^{n}\right\| ^2+\left\| \psi ^{n,j}\right\| ^2\right) \nonumber \\ & \le C_6\sum _{j=1}^J\left( \left\| \Delta w_0^{n}\right\| ^2+C_2\sum _{i=1}^q\left\| \Delta w_i^{n}\right\| ^2\right) \nonumber \\ & \le JC_6(1+C_2)\left[ \left\| \Delta w_0^{n}\right\| ^2+\sum _{i=1}^q\left\| \Delta w_i^{n}\right\| ^2\right] \nonumber \\ & \le JC_6(1+C_2)\eta ^2(1+\alpha )^2\left\| E_{W}(W^n)\right\| ^2 \nonumber \\ & = C_7\eta ^2(1+\alpha )^2\left\| E_W(W^n)\right\| ^2 \end{aligned}$$

where $$C_6=C_3\max \{C_2^2,C_5^2\}$$, $$C_7= JC_6(1+C_2)$$

Similarly, we can evaluate $|\delta _3|$ as follows:

31 $$\begin{aligned} |\delta _3| & \le \frac{C_3}{2}\sum _{j=1}^J\left( \left\| \Delta w_0^{n}\right\| ^2+\left\| \psi ^{n,j}\right\| ^2\right) \nonumber \\ & \le \frac{C_3}{2}\sum _{j=1}^J\left( \left\| \Delta w_0^{n}\right\| ^2+C_2\sum _{i=1}^q\left\| \Delta w_i^{n}\right\| ^2\right) \nonumber \\ & \le {\frac{1}{2}}JC_3(1+C_2)\left[ \left\| \Delta w_0^{n}\right\| ^2+\sum _{i=1}^q\left\| \Delta w_i^{n}\right\| ^2\right] \nonumber \\ & \le {\frac{1}{2}}JC_3(1+C_2)\eta ^2(1+\alpha )^2\left\| E_{W}(W^n)\right\| ^2\nonumber \\ & = C_8\eta ^2(1+\alpha )^2\left\| E_{W}(W^n)\right\| ^2 \end{aligned}$$

where $$C_8={\frac{1}{2}}JC_3(1+C_2)$$.

Similarly, we obtain

32 $$\begin{aligned} |\delta _1| & \le {\frac{1}{2}}JC_3^2C_4^2C_5\sum _{i=1}^q \left\| \Delta w_i^n\right\| ^2\nonumber \\ & \le {\frac{1}{2}}JC_3^2C_4^2C_5\eta ^2(1+\alpha )^2\sum _{i=1}^q\left\| E_{w_{i}}(W^n)\right\| ^2\nonumber \\ & = C_9\eta ^2(1+\alpha )^2\left\| E_{W}(W^n)\right\| ^2 \end{aligned}$$

where $$C_9={\frac{1}{2}}JC_3^2C_4^2C_5$$.

Using $C_1=C_7+C_8+C_9$, from (29)–(32) and (*A*3) we can obtain

33 $$\begin{aligned} E(W^{n+1})-E(W^{n}) & \le -\eta (1-\lambda \eta (1+\alpha )^2M)\sum _{i=1}^q \sum _{k=0}^p\left( E_{w_{ik}}(W^n)\right) ^2 +\delta _1+\delta _2+\delta _3\nonumber \\ & \le & \le -\eta (1-M\lambda \eta (1+\alpha )^2)\left\| E_{W}(W^n)\right\| ^2\nonumber \\&+c_1\eta ^2(1+\alpha )^2\left\| E_{W}(W^n)\right\| ^2\nonumber \\ &= -\eta (1-M\lambda \eta (1+\alpha )^2-C_1\eta (1+\alpha )^2)\left\| E_{W}(W^n)\right\| ^2\nonumber \\ & \le 0 \end{aligned}$$

There holds $$E(W^{n+1})\le E(W^n)$$ (n = 1,2,...). $$\square$$

**Step 3.** we show $\lim \nolimits _{n\rightarrow \infty }\Vert E_W (W^n)\Vert =0.$

### *Proof*

From Step 2, we know that the nonnegative sequence $\{E(W^n)\}$ is monotone and it is also bounded. Hence, there must exist $E^*\ge 0$ such that $\lim \nolimits _{k\rightarrow \infty }E(W^n)=E^*$.

Taking $\beta =\eta -(M\lambda +C_1)\eta ^2(1+\alpha )^2$, it follows from Assumption (*A*3) that $\beta >0$. $\sigma ^n=\sum _{i=1}^q \sum _{k=0}^p(E_{w_{ik}}(W^n))^2=\Vert E_W(W^n)\Vert ^2$ and using (33), we get

$$\begin{aligned} E(W^{n+1}) & \le E(W^{n})-\beta \left\| E_W(W^n)\right\| ^2\\ & \le \cdots \\ & \le E(W^0)-\beta \sum _{k=0}^n \left\| E_W(W^k)\right\| ^2 \end{aligned}$$

Since $E(W^{n+1})\ge 0$, it gives that

$$\begin{aligned} \beta \sum _{k=0}^n\left\| E_W(W^k)\right\| ^2\le E(W^0) \end{aligned}$$

Let $n\rightarrow \infty$, it holds that

$$\begin{aligned} \sum _{k=0}^\infty \left\| E_W(W^k)\right\| ^2\le \frac{1}{\beta }E(W^0)<\infty \end{aligned}$$

This results in

$$\begin{aligned} \lim \limits _{n\rightarrow \infty }\left\| E_W(W^n)\right\| ^2=0 \end{aligned}$$

Thus

34 $$\begin{aligned} \lim \limits _{n\rightarrow \infty }\left\| E_W (W^n)\right\| =0. \end{aligned}$$

$$\square$$

**Step 4.** Add the assumption (*A*4), we show $\lim \nolimits _{n\rightarrow \infty }(W^n)=W^*,$ where $W^*\in \Phi _0$.

### *Proof*

Note that the error function *E*(*W*) defined in (11) is continuous and differentiable. According to (21) and (34), we get

$$\begin{aligned} \sum _{i=1}^q \sum _{k=0}^p(\Delta w_{ik}^n)^2\le \left\| E_W(W^n)\right\| ^2 \end{aligned}$$

i.e.

35 $$\begin{aligned} \lim \limits _{n\rightarrow \infty } \left\| W^{n+1}-W^n\right\| =0 \end{aligned}$$

According to the assumption (*A*4), (34), (35) and Lemma 1, there exists a point $W^*\in \Phi _0$, such that

36 $$\begin{aligned} \lim \limits _{n\rightarrow \infty }W^n=W^*. \end{aligned}$$

$$\square$$

Now, we proved the Theorem 1 by Step 1–Step 4.
